# The Effects of Hot Isostatic Pressing (HIP) and Heat Treatment on the Microstructure and Mechanical Behavior of Electron Beam-Melted (EBM) Ti–6Al–4V Alloy and Its Susceptibility to Hydrogen

**DOI:** 10.3390/ma17122846

**Published:** 2024-06-11

**Authors:** Noa Lulu-Bitton, Nissim U. Navi, Shlomo Haroush, Eyal Sabatani, Natalie Kostirya, Eitan Tiferet, Yaron I. Ganor, Ofer Omesi, Gennadi Agronov, Noam Eliaz

**Affiliations:** 1 Department of Materials Science and Engineering, Tel-Aviv University, Tel Aviv 6997801, Israel; noabitton@mail.tau.ac.il (N.L.-B.); eyal.sabatani@gmail.com (E.S.); 2 Nuclear Research Center Negev (NRCN), Beer Sheva 84190, Israel; monih6655@gmail.com (S.H.); natalie.kostirya25@gmail.com (N.K.); tiferete@gmail.com (E.T.); yarong@rotemi.co.il (Y.I.G.); ofero101969@gmail.com (O.O.); gidiaz@walla.com (G.A.); 3 AM Center, Rotem Industries Ltd., Mishor Yamin 86800, Israel

**Keywords:** additive manufacturing (AM), electron beam melting (EBM), Ti–6Al–4V alloy, titanium hydride, hydrogen embrittlement (HE), small punch test (SPT), hot isostatic pressing (HIP), heat treatment

## Abstract

The effects of the secondary processes of Hot Isostatic Pressing (HIP) at 920 °C and Heat Treatment (HT) at 1000 °C of Electron Beam-Melted (EBM) Ti–6Al–4V alloy on the microstructure and hydrogen embrittlement (HE) after electrochemical hydrogen charging (EC) were investigated. Comprehensive characterization, including microstructural analysis, X-ray diffraction (XRD), thermal desorption analysis, and mechanical testing, was conducted. After HIP, the β-phase morphology changed from discontinuous Widmanstätten to a more continuous structure, 10 times and ~1.5 times larger in length and width, respectively. Following HT, the β-phase morphology changed to a continuous “web-like” structure, ~4.5 times larger in width. Despite similar mechanical behavior in their non-hydrogenated state, the post-treated alloys exhibit increased susceptibility to HE due to enhanced hydrogen penetration into the bulk. It is shown that hydrogen content in the samples’ bulk is inversely dependent on surface hydride content. It is therefore concluded that the formed hydride surface layer is crucial for inhibiting further hydrogen penetration and adsorption into the bulk and thus for reducing HE susceptibility. The lack of a hydride surface layer in the samples subject to HIP and HT highlights the importance of choosing secondary treatment process parameters that will not increase the continuous β-phase morphology of EBM Ti–6Al–4V alloys in applications that involve electrochemical hydrogen environments.

## 1. Introduction

Titanium alloys are common in a variety of applications, such as aviation, space applications [1], and bone implants [2,3], thanks to their high specific strength and high corrosion resistance in seawater and in the human body environment. The α(hcp) + β(bcc) Ti–6Al–4V alloy stands as the predominant Ti-based alloy. Modifying its microstructure through thermo-mechanical procedures profoundly influences its mechanical behavior and resistance to corrosion [4,5,6,7,8,9,10].

Ti-based alloys have been traditionally manufactured by processes such as casting, machining, forging, and powder metallurgy (P/M). In recent decades, additive manufacturing (AM) was added thanks to advantages such as the manufacturability of complex shapes and near-net-shape production of personalized designs [11]. The microstructure resulting from AM processes is different, mainly due to the repeated thermal cycles and fast cooling rates [12,13,14], thus affecting the properties of the AM material. Lewandowski and Seifi [15] compared the mechanical properties of AM Ti–6Al–4V with those of forged, wrought, and annealed alloys. While the AM alloys had equal or higher yield strength and ultimate tensile strength compared to the others, the former exhibited smaller elongation. AM processes are usually combined with thermal post-processing steps to attain the mechanical properties required for the product [16,17]. Ganor et al. [18] studied the influence of secondary processes, such as hot isostatic pressing (HIP) and heat treatment (HT), on the microstructure and mechanical properties of electron beam-melted (EBM) Ti–6Al–4V. These secondary processes resulted in an increase in the β-phase content. HIP at 780 °C preserved the as-built microstructure, yield stress, and ultimate tensile strength (UTS) and improved the elongation and fatigue resistance. HIP at 920 °C, on the other hand, resulted in a decrease in yield stress and UTS, but improved the elongation and fatigue resistance. HT treatments at 780 and 1000 °C resulted in a decrease in yield stress and UTS, but improved the elongation. Pushilina et al. [19] investigated the influence of HT at 750 °C for 1 h in vacuum on the microstructure, mechanical properties, and hydrogen sorption rate of EBM Ti–6Al–4V. HT was found to decrease the microhardness, strength, and plastic characteristics. Illsley et al. [20] explored the mechanical behavior of as-built EBM Ti–6Al–4V subject to HIP following casting and forged conditions. The EBM alloy exhibited the lowest load and displacement values. Across all alloys, a mixed-mode fracture was observed, featuring a combination of ductile and brittle behavior.

Hydrogen alters the mechanical behavior of metallic materials, including titanium alloys, e.g., due to hydrogen embrittlement (HE) as a result of hydride formation [21,22]. The susceptibility of an alloy to hydrogen may be affected by the hydrogen charging method (e.g., electrochemical charging (EC) vs. gaseous charging (GC)), charging conditions, exposure time, and microstructure [23,24]. HE in Ti–6Al–4V alloys depends on the morphology of the α and β phases and on the α/β interphases. The β phase is characterized by higher diffusivity and solubility of hydrogen [25,26,27,28,29]. Thus, high β-phase content in the alloy will enhance hydrogen diffusion through the β-phase, which may eventually favor hydride formation around α/β interphase boundaries. This mechanism was well demonstrated by Kim et al. [30,31,32]. In addition, the dissolution of hydrogen in Ti alloys results in lattice parameter expansion in both α and β phases, leading to induced strain [26]. The reaction of hydrogen with matrix elements can form hydride phases such as δ (fcc), γ (fct, *c*/*a* > 1), and ε (fct, *c*/*a* < 1) [26,27,28,29,33,34,35,36,37]. Several studies were conducted to explore the effect of the typical microstructures of AM Ti–6Al–4V on its sensitivity to HE [19,28,29,36,38,39,40,41,42,43,44,45]. Navi et al. [41] investigated the influence of EC on both EBM and wrought Ti–6Al–4V alloys with a similar β-phase contents. It was shown that the EBM alloy exhibited greater susceptibility to HE compared to its counterpart wrought alloy. It was further shown that a higher density of the α/β interphase boundaries and the discontinuous arrangement of β-phase in the EBM alloy facilitated the formation of hydrides, microvoids, and cracking along the interphase boundaries. Brosh et al. [46] associated the occurrence of microvoids with hydride precipitation from a hydrogen-enriched phase. In addition, it was found that the surface of the hydrogenated alloys consisted of α_H_ and β_H_ solid solutions and δ_a_ and δ_b_ (fcc) hydrides, which are variants of the δ-hydride (TiH_x_) [41]. The mechanical properties of AM Ti–6Al–4V alloy are significantly affected by exposure to hydrogen. Kacenka et al. [47] showed that the tensile properties of selective laser melted (SLM) Ti–6Al–4V alloy were inferior after EC compared to those of a wrought alloy. They attributed the differences to a higher amount of hydrogen intake in the SLM alloy. Wu et al. [48] investigated the influence of EC on the microstructure and tensile properties of Ti–6Al–4V alloy manufactured by the SLM process. It was found that the texture of the AM alloy had a significant impact on its mechanical properties and susceptibility to HE. The microstructure of the hydrogenated alloy consisted of δ-TiH_2_ and δ-TiH_x_ hydrides, as well as a solid solution of hydrogen in the α-phase. Following hydrogenation, both the tensile and yield strength increased by roughly 15%, while the UTS and elongation decreased by 25% and 67%, respectively. The fracture morphology of the as-charged specimen was representative of a fully lamellar microstructure, exhibiting a continuous β-phase. Furthermore, microcracks developed in the α-phase or along the α/β interphase boundary, and a sharp ductile-to-brittle transition occurred after charging.

Metalnikov et al. [40] investigated the impact of both GC and EC on EBM Ti–6Al–4V compared to the wrought alloy. In the EBM alloy, hydrides were formed following either EC (γ-TiH and δ-TiH_x_) or GC (δ-TiH_x_). No cracks were observed in the EBM alloy after hydrogenation, while severe cracking was observed in the wrought alloy.

In our previous studies [23,24,41], we showed that following EC, the EBM Ti–6Al–4V alloy displayed higher susceptibility to HE compared to the wrought alloy, despite both alloys containing approximately 6 wt% β and similar impurity levels. This disparity was attributed to microstructural variations favoring the formation of a surface layer enriched with δ_a_ and δ_b_ hydrides in the wrought alloy, thereby impeding hydrogen ingress into the bulk material [23,41]. Lulu-Bitton et al. showed that following GC at high temperature, the hydrogen effect was less dependent on the microstructure; hence, the two alloys exhibited similar mechanical degradation [24] based on small punch tests (SPTs). The SPT technique was reviewed in detail in our previous reports [23,24], as well as by others [49,50,51,52,53,54], and will therefore not be described in detail herein.

It was shown above that secondary processes such as HIP and HT are expected to alter the phase morphology and content. They are thus expected to affect the alloy’s susceptibility to HE and mechanical degradation. Yet, we could find only one report [55] of the effect of secondary processes on the mechanical behavior of electrochemically hydrogenated SLM Ti–6Al–4V alloy and no report on the mechanical behavior of electrochemically hydrogenated EBM Ti–6Al–4V after secondary processes.

The main goal of the current study is thus to reveal the influence of HIP and HT secondary processes on the microstructure and mechanical behavior of EBM Ti–6Al–4V before and after EC. Comprehensive characterization, including microstructural analysis, X-ray diffraction (XRD), thermal desorption analysis, and SPT mechanical testing, was employed.

## 2. Materials and Methods

### 2.1. EBM of Ti–6Al–4V and Secondary Processes

**Figure 1:**
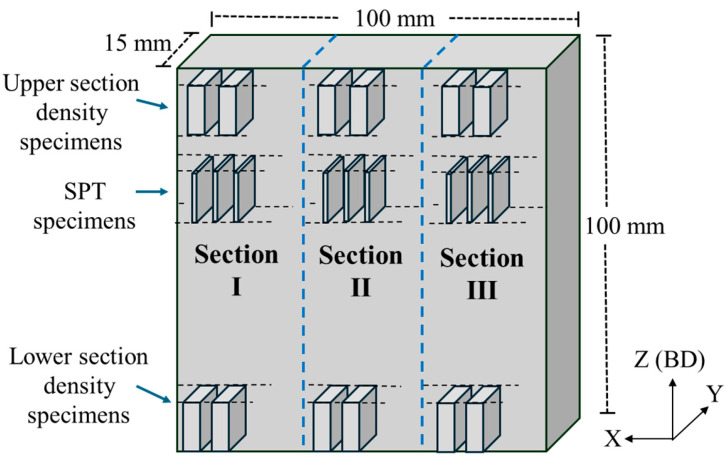
Schematic illustration of the extraction of specimens for density and SPT measurements. BD = build direction.

A rectangular sample of Ti–6Al–4V with dimensions of 15 × 100 × 100 mm^3^ was manufactured via EBM at the AM Center of Rotem Industries Ltd. (Mishor Yamin, Israel) utilizing an Arcam Q20 Plus EBM machine (Arcam, Mölndal, Sweden) and using a standard Grade 5 spherical powder with a size distribution of 45–106 µm. Detailed EBM printing parameters can be found in [41]. The rectangular box was cut by electric discharge machining (EDM) into three identical sections, as illustrated in Figure 1. The long edge of the three sections was orientated in the Z build direction. One section was kept in the as-built condition, the second underwent HIP treatment at 920 °C for 2 h under a pressure of 120 MPa in Ar [18], in compliance with ASTM F3301 [56]. The heating and cooling rates for the HIP process were set at 4 °C/min. The third section underwent HT (annealing) at 1000 °C for 2 h under a controlled vacuum environment (10^−3^ mbar) [18]. The heating rate was 10 °C/min, whereas cooling was done by furnace coold. All process parameters are summarized in Table 1.

Samples for EC and SPT were cut by EDM in the Z direction to a thickness of 0.7 mm, grinded to a thickness of 0.5 mm, and then polished on both sides down to 1 μm. Samples for density measurement prior to hydrogenation were cut from the upper and lower sections to a thickness of 4 mm each and were mechanically ground down to 1200-grit SiC paper.

### 2.2. Electrochemical Charging (Hydrogenation)

**Figure 2:**
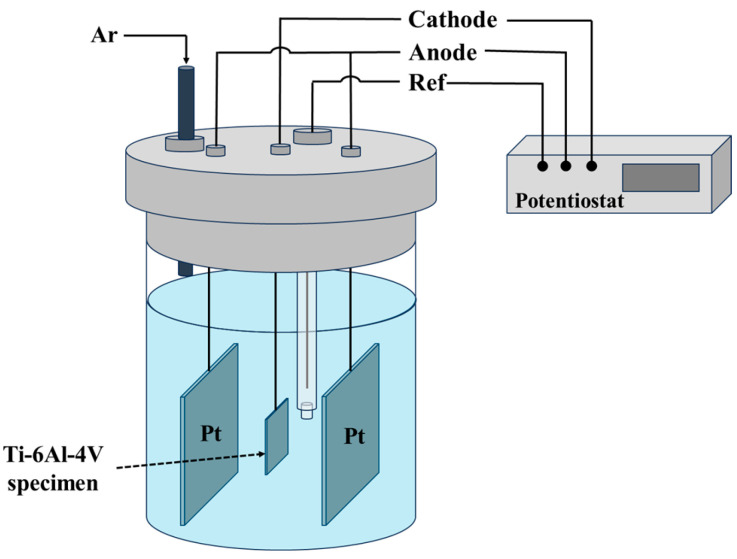
Schematic illustration of the EC cell setup.

EC was performed galvanostatically (*i* = −7.5 mA/cm^2^) at room temperature (RT) in a three-electrode cell for 8 h. The sample being tested was used as the cathode and was positioned between two parallel platinum foil anodes, as illustrated in Figure 2. Ag/AgCl was used as a reference electrode. H_3_PO_4_–glycerin (1:2 volume ratio) was used as an electrolyte [23,41,45,46,57,58]. Due to its high viscosity, this electrolyte is known to facilitate hydrogen adsorption onto the metal surface and increase the cathodic charging efficiency [21,58]. Before EC, the electrolyte was purged with Ar for 30 min. Ar flow was maintained above the electrolyte during charging to prevent oxygen absorption. Fresh electrolyte was employed for each charging experiment.

### 2.3. Material Characterization

A comprehensive description of the microstructure, SPT, and fractographic characterization is provided in [23,24], while a detailed description of chemical analysis is provided in [23,41]. Therefore, herein we will describe them only briefly.

#### 2.3.1. Chemical Composition and Density

Carbon, oxygen, nitrogen, and hydrogen level characterization was performed by the procedure described in [23,41]. Three specimens were analyzed as triplicates for each element. The chemical composition of key elements in the EBM alloy was confirmed using energy-dispersive X-ray spectroscopy (EDS). Density measurement was carried out via the Archimedes method using deionized (DI) water at room temperature (RT) [59]. The density measurements were conducted using six specimens from each alloy, three from the lower section and three from the upper section (Figure 1).

#### 2.3.2. Microstructural Analysis by Scanning Electron Microscopy (SEM) and X-ray Diffraction (XRD)

Microstructural characterization was performed using SEM (Quanta 200 FEG ESEM, FEI, MA, USA) coupled with an EDS detector (Oxford X-Max SDD, Abington, UK). The sample preparation procedure for the metallographic characterization examination is thoroughly described in [24]. SEM micrographs were analyzed using the ImageJ freeware program version 1.54d [60] to assess the β-phase length and width.

RT-XRD was applied for phase identification, phase content assessment, and lattice parameter determination. XRD patterns were obtained from the sample surface using an Empyrean powder diffractometer (Panalytical B.V., Almelo, The Netherlands) equipped with an X’Celerator position-sensitive detector (Panalytical B.V., Almelo, The Netherlands). The scan was performed within the range of 2θ = 20–90 degrees, although the data presented herein are narrowed to 2θ = 35–45 degrees to emphasize major changes. The test procedure parameters are described in detail in [24]. Lattice parameters and phase content were refined using Rietveld analysis with TOPAS software, version 5 (Bruker AXS, Madison, WI, USA).

#### 2.3.3. Thermal Desorption Analyses

The total hydrogen content in the alloys was assessed utilizing a homemade temperature-programmed desorption mass spectrometry (TPD-MS) apparatus [61]. TPD data analysis procedures are explained in [57,62]. The use of TPD with respect to hydrogenated Ti-based alloys is reported in [36,41,63,64,65,66].

Following SPT, TPD measurements were performed on three samples for each charging condition, each sample with a mass of ~20 mg. The TPD apparatus was programmed to raise the temperature from RT to 800 °C at a constant rate of 10 °C/min under a 50 mL/min flow of high-purity He. Additional test procedure information can be found in [23].

#### 2.3.4. Mechanical Testing by SPT

The SPT experiments were conducted at RT using homemade apparatus. The tested samples were as-AM, after-HIP, and after-HT, before and after hydrogenation. Three measurements were carried out for each charging condition. Each test was done in three major steps: (1) clamping of the specimen between the lower and upper dies under 2500 N, (2) pre-loading the specimen up to 30 N and balancing the stoke transducer (Instron LVDT), and (3) pushing the ball into the specimen under stroke control at a speed of 0.1 mm/min up to failure, i.e., the end test criterion was a sharp load drop [23,51,67]. A 2.4 mm diameter hard steel ball was used. The rectangular specimen dimensions were 10 × 15 mm^2^ and the thickness was 0.5 mm. Three measurements were carried out at the sample center for each charging condition. SEM analysis was employed to determine the fracture mode upon specimen failure.

## 3. Results and Discussion

### 3.1. Chemical Composition and Density

The concentrations of the light elements (C, H, O, and N) in the non-hydrogenated as-built EBM Ti–6Al–4V, as well as after HIP and HT, are listed in Table 2 (weight parts per million, wppm). It can be seen that oxygen is the predominant impurity in all three alloys and that the total impurity content is consistent among all alloys, with a slight increase observed in the HT-treated alloy. The chemical composition of the major elements obtained by EDS analysis in the as-built alloy was 89.9 Ti, 5.9 Al, and 4.2 V (values in wt%), aligning closely with the ASTM F2924 specification [68]. This indicates that no contamination was introduced into the alloys during the secondary processes.

The average density of the non-hydrogenated alloy in the as-built, HIP, and HT conditions was 4.415 ± 0.003 (99.59%), 4.422 ± 0.002 (99.75%), and 4.416 ± 0.002 (99.62%) g/cm^3^, respectively, while the theoretical density was 4.4328 g/cm^3^ [22,26,27,42]. This indicates a slight density improvement for the HIP alloy. These trends are in agreement with the trends observed by Ganor et al. [18]. The density difference between the lower and upper sections was less than 0.002 g/cm^3^, indicating process stability along the build direction.

### 3.2. Microstructure

**Figure 3:**
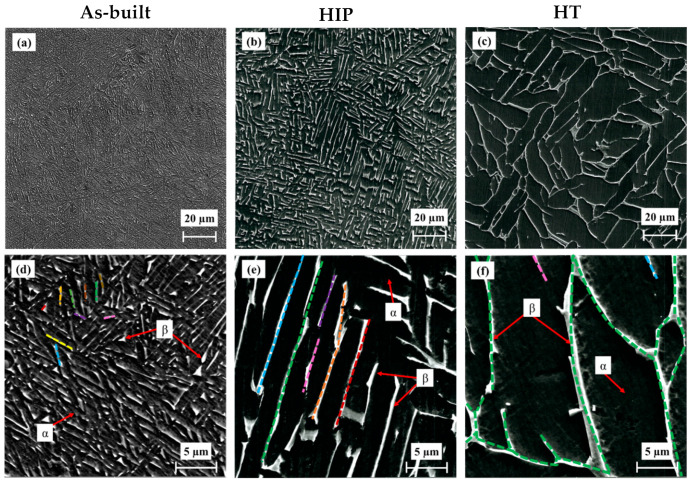
Cross-sectional SEM secondary electron (SE) micrographs of non-hydrogenated as-built (**a**,**d**), HIP (**b**,**e**), and HT alloys (**c**,**f**). The β-phase continuity in the three alloys is illustrated by the colored lines in (**d**–**f**).

SEM micrographs of the non-hydrogenated EBM Ti–6Al–4V alloy are shown in Figure 3. Figure 3a,d shows the as-built alloy, Figure 3b,e shows the HIP alloy, and Figure 3c,f shows the HT alloy. The microstructure of the as-built alloy consists of two phases—α (darker, major) and β (brighter, minor). Our previous EDS results in [18,23,24,69] revealed that the darker α phase has higher Al content (Al stabilizes the α-phase), whereas the brighter β phase has higher vanadium content (V stabilizes the β-phase). One can see that the β-phase morphology is discontinuous as a result of rapid solidification during the EBM process, which generates a large density of α/β interphases (Figure 3d). The α/β morphology is typical of EBM Ti–6Al–4V, namely a Widmanstätten (basket weave) structure, where the β-phase laths are lying along the α-phase boundaries [18,24,28,41,69]. After HIP (Figure 3b,e), the β-phase morphology changed to longer and wider lamellas, more continuous than the as-built alloy. Following HT (Figure 3c,f), the microstructure of the β phase seems to be longer, more continuous, and wider than the β phase in the HIP alloy. These results are in agreement with those of Ganor et al. [18].

The level of discontinuity of the β phase was estimated by measuring the length of at least 20 β lamellas from several SEM metallographic images. This is illustrated by different color markings of individual β-phase laths (Figure 3d–f). It was found that the average length and median of the β-phase laths in the as-built alloy were 1.8 ± 0.7 µm and 1.5 µm, respectively, while for the HIP alloy they were 18.4 ± 7.5 µm and 17.5 µm, respectively (i.e., ~10 times greater). The β-phase lamellas in the HT alloy are continuous across the specimen’s cross-section, forming a “web-like” morphology.

The average width and median values of the β phase in the as-built alloy were 0.28 ± 0.13 µm and 0.25 µm, respectively; in the HIP alloy they were 0.41 ± 0.07 µm and 0.42 µm, respectively; and in the HT alloy they were 1.25 ± 0.37 µm and 1.16 µm, respectively.

The major changes in length, width, and continuity of the β phase after the secondary processes, compared to the as-built alloy, can be explained by the relatively higher process pressure and temperature of the HIP treatment, and a temperature of 1000 °C, which is above the β transus temperature for the HT (~995 °C) [6,18], combined with the sufficient dwell time that characterizes both processes.

**Figure 4:**
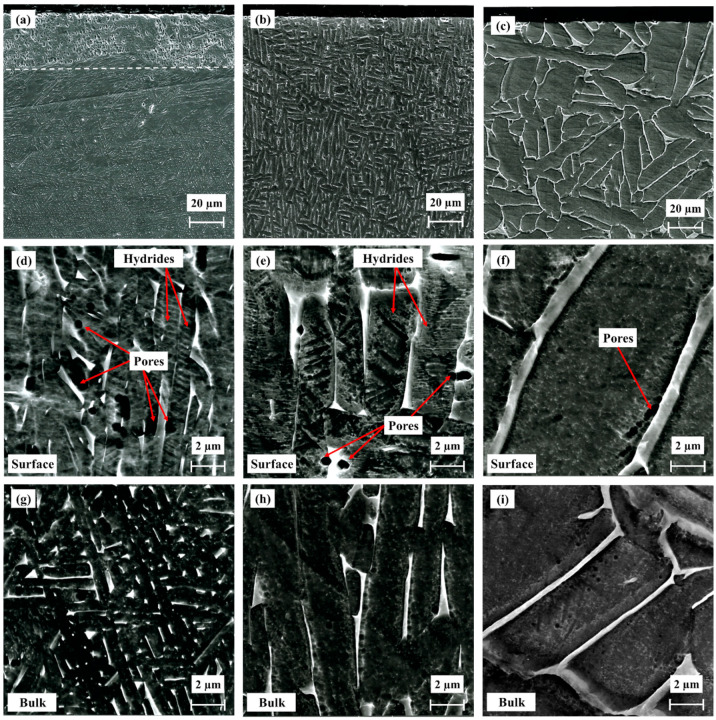
Cross-sectional SEM SE micrographs of hydrogenated EBM alloy (**a**,**d**,**g**), EBM and HIP (**b**,**e**,**h**), and EBM and HT (**c**,**f**,**i**).

The microstructure of the alloys after hydrogenation is presented in Figure 4. The hydrogenated as-built alloy, shown in Figure 4a,d,g, contains a surface layer with a thickness of ~35 µm (marked by white dashed line in Figure 4a), as first reported for electrochemically hydrogenated Ti–6Al–4V by Navi et al. [41] and then by Lulu-Bitton et al. [23]. All hydrogenated alloys contain pores (micro-voids), as reported in [23,41,46]. The micro-voids are located along the α/β interphase boundaries, as shown in Figure 4d–f, and are less common in the bulk (Figure 4g–i). Brosh et al. [46] suggested a mechanism of void formation due to the EC of Ti–6Al–4V alloy, which was first reported in [41], according to which volume shrinkage results from hydride precipitation from a supersaturated β_H_ phase. The major difference in the microstructures of the hydrogenated HIP (Figure 4b,e,h) and HT (Figure 4c,f,i) alloys is that they do not contain a surface hydride layer, in contrast to the as-built alloy. This difference can be attributed to a higher content of, as well as wider and more continuous, β phase in the post-treated alloys, which acts as a continuous pathway for hydrogen diffusion into the bulk [44,70] via a wide range of trajectories and thus inhibits hydrogen build-up and hydride layer formation at the alloy’s surface.

### 3.3. XRD Phase Analysis

#### Phase Content and Lattice Parameter

**Figure 5:**
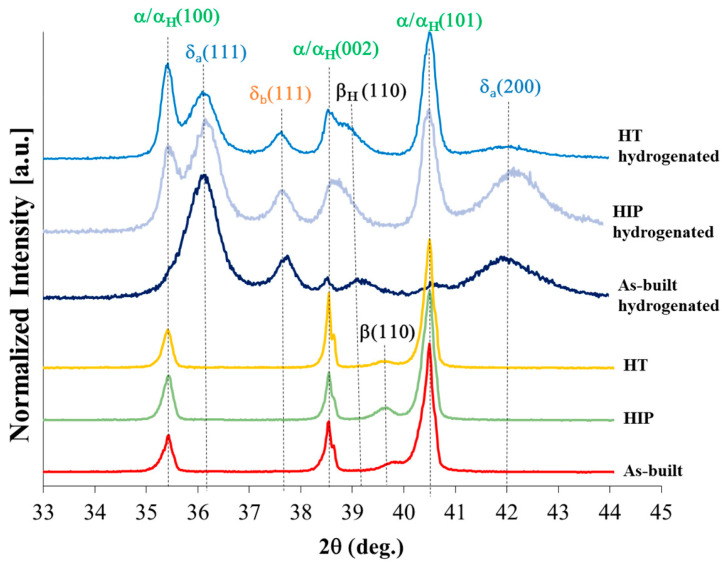
XRD patterns of non-hydrogenated and hydrogenated EBM Ti–6Al–4V alloys in the as-built, HIP, and HT conditions.

**Figure 6:**
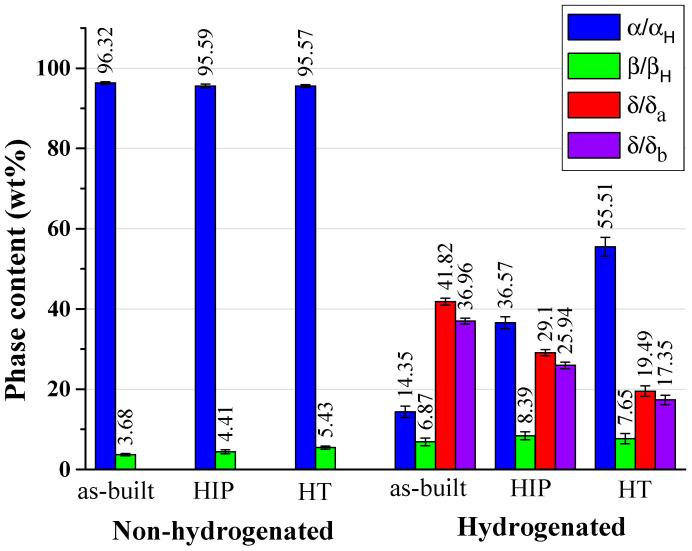
Phase content (wt%) in the non-hydrogenated and hydrogenated alloys (error bars calculated by Rietveld refinement).

Normalized XRD patterns (maximal peak is 100%) of the non-hydrogenated and hydrogenated alloys are shown in Figure 5 (the ordinate values are after arbitrary scaling). Figure 6 and Table 3 summarize the phase content and lattice parameters, respectively, as calculated by TOPAS Rietveld refinement (with all *R*_wp_ values below 14.7).

For the non-hydrogenated alloys, the content of β phase was similar, with a slight increase in the β-phase content after HIP and HT secondary processes (from 3.68 to 4.41 and 5.43 wt%, respectively). The slight increase in the β-phase content after the secondary processes is probably due the high temperature, applied pressure, and dwell time, as discussed above.

The α phase shows a slight decrease after the secondary processes, following the β-phase slight increase. These results are in agreement with the trends observed by Ganor et al. [18]. It should be noted that no major changes were noticed in the lattice parameters of the α phase following secondary processes, and hence in the α-phase *c*/*a* ratio. The lattice parameters of the β phase were increased by more than 0.5% due to the secondary processes.

After hydrogenation, all alloys contained α/α_H_ (the two α phases cannot be distinguished in the XRD analysis), β_H_ solid solutions, and δ_a_ and δ_b_ hydrides. The δ_a_ and δ_b_ hydrides are based on the δ (TiH_x_) phase with an fcc structure, space group Fm-3m (225), and were previously identified by Navi et al. and then by others [23,41,44,45,46,48,69]. The content of δ_a_ + δ_b_ hydrides are 79 wt% in the as-built, 55 wt% in the HIP, and 37 wt% in the HT condition.

The lattice parameter *a* of the α/α_H_ phase in all conditions did not change significantly after hydrogenation. The α/α_H_ content in the as-built, HIP, and HT condition is reduced after hydrogenation to 14.4, 36.6, and 55.5 wt%, respectively. This reduction is in agreement with the results in [23,41,69].

All hydrogenated alloys showed a relative increase in the β_H_ phase content (almost twofold) compared to their initial β values, with a similar content of 6.9, 8.4, and 7.6 wt% in the as-built, HIP, and HT conditions, respectively. Similar results for the as-built condition following EC were reported by Lulu-Bitton et al. [23]. All lattice parameters of the β_H_ phase show a ~2% increase compared to the original non-hydrogenated β-phase due to the high solubility of hydrogen in the β_H_ solid solution, in contrast to its poor solubility in the α-phase [71,72].

### 3.4. TPD Analysis

**Figure 7:**
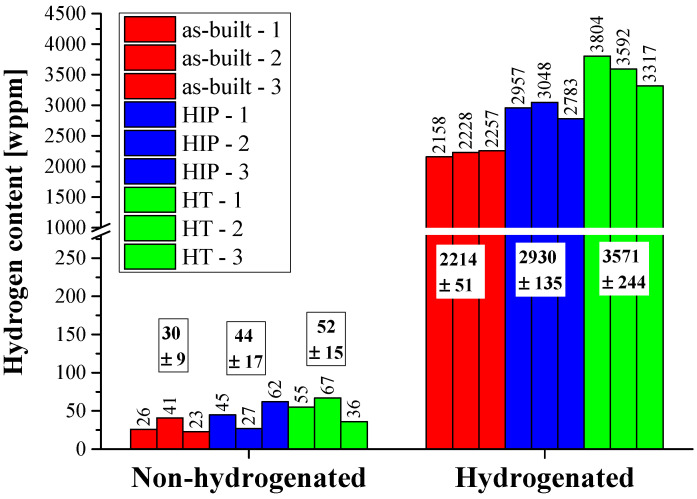
Total amount of desorbed hydrogen in the as-built (red), HIP (blue), and HT (green) non-hydrogenated and hydrogenated alloys.

The TPD results are presented in Figure 7. It can be seen that the initial hydrogen content in all three non-hydrogenated alloys is low and similar, 23–67 (wppm). After hydrogenation, the hydrogen content in each alloy increases significantly, with the smallest amount of desorbed hydrogen in the as-built alloy and the highest amount in the HT alloy.

**Figure 8:**
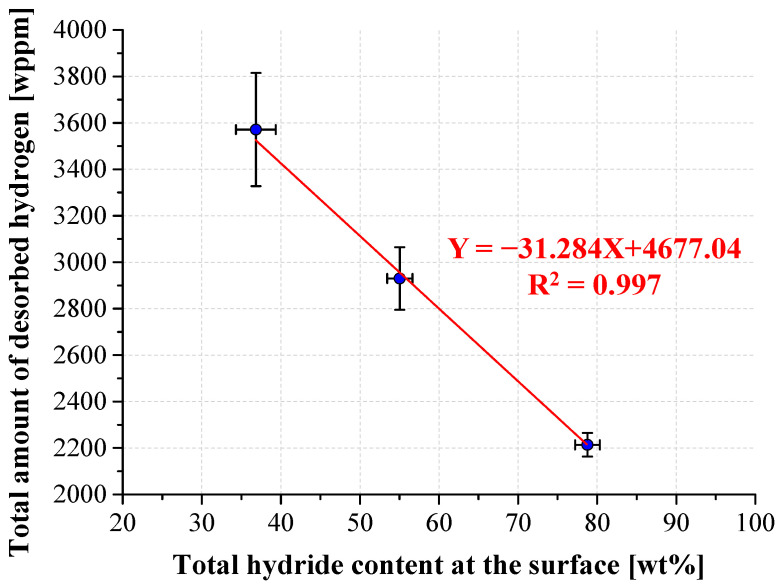
Total amount of desorbed hydrogen from hydrogenated as-built, HIP, and HT alloys as a function of the total hydride content at the surface.

The total amount of desorbed hydrogen in the as-built, HIP and HT hydrogenated alloys, as a function of total hydride content (δ_a_ + δ_b_), which represents the total hydride phases in the surface of the alloys, is shown in Figure 8 (it was shown above that the content of the β_H_ phase in all hydrogenated alloys is similar and thus does not expect to have a major influence on the total hydrogen content). It can be seen from Figure 8 that an inverse correlation exists between the hydride content at the surface and the total hydrogen uptake. However, one would expect a linear correlation between the hydride content and the total amount of desorbed hydrogen. This apparent discrepancy is attributed to the original, non-hydrogenated, β-phase morphology of the alloys. In the hydrogenated as-built alloy, a hydride surface layer was prominent, mainly due to the discontinuous Widmanstätten morphology of the original β phase that characterized the as-built alloy. This surface layer inhibits hydrogen permeation into the bulk [23,73], and therefore this alloy shows the lowest hydrogen amount. The hydrogenated HIP alloy, which contains more hydrogen than the as-built hydrogenated alloy, contains more continuous β-phase morphology, which increases hydrogen uptake efficiency to the bulk. The hydrogenated HT alloy, which contains the largest amount of hydrogen, is characterized with continuous “web-like” β-phase morphology, which further increases hydrogen uptake efficiency in the bulk. The high efficiency hydrogen uptake to the bulk in both post-treated alloys explains the lack of hydride layer formation in both surfaces.

### 3.5. SPT Curves

**Figure 9:**
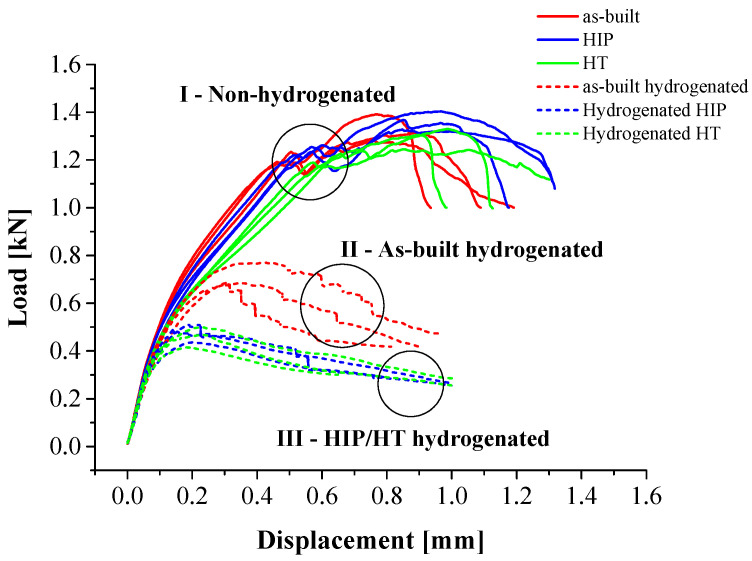
SPT curves of non-hydrogenated and hydrogenated EBM Ti–6Al–4V: as-built (red), HIP (blue), and HT (green).

The load-displacement curves of the non-hydrogenated and hydrogenated alloys are shown in Figure 9. It is apparent that the mechanical behavior can be classified into three distinct groups: (I) all alloys prior to hydrogenation, (II) hydrogenated as-built alloy, and (III) hydrogenated HIP and HT alloys. Hydrogen charging substantially degrades the mechanical behavior of all alloys (groups II and III), as expected [23,47]. However, it can be seen that HIP and HT post-treated alloys degrade more than the as-built alloy. A similar trend for the EBM as-built alloy following EC was reported by Lulu-Bitton et al. [23].

**Figure 10:**
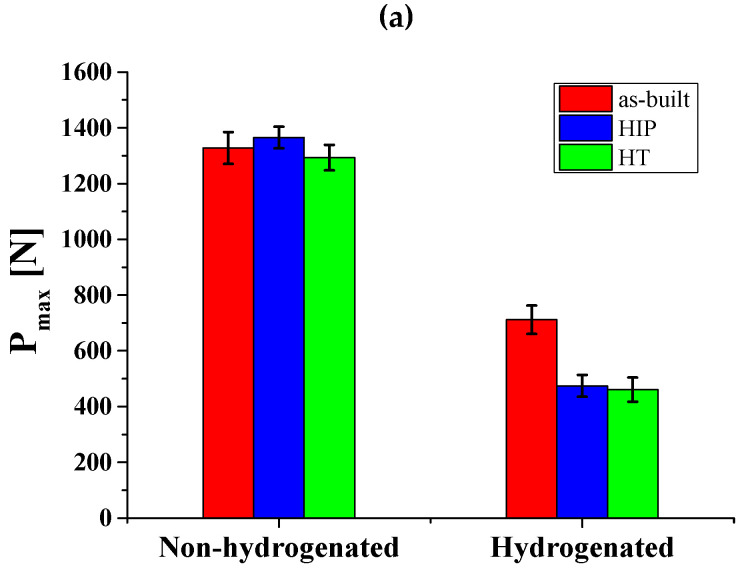

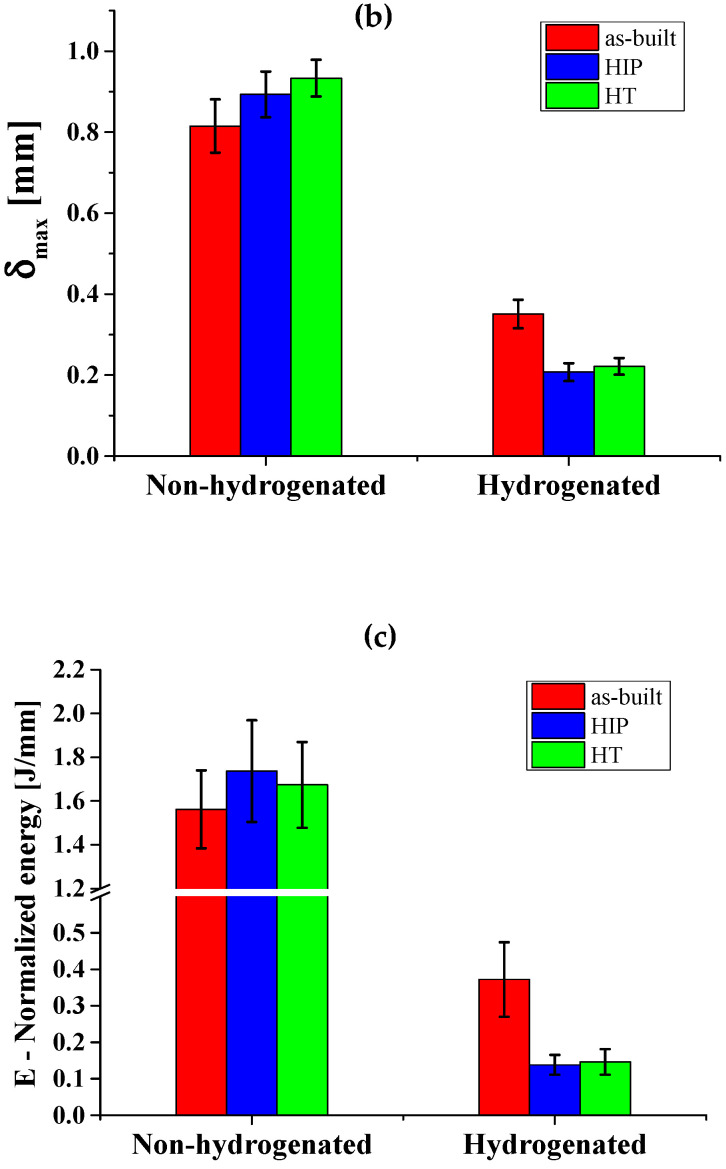
(**a**) Maximum load, *P*_max_. (**b**) Displacement at maximum load, *δ*_max_. (**c**) Normalized energy from SPT curves of as-built, HIP, and HT non-hydrogenated and hydrogenated alloys.

The mechanical behavior of the non-hydrogenated and hydrogenated alloys, based on SPT curves, is summarized in Table 4 and Figure 10. Figure 10a shows the maximum load (*P*_max_) obtained for the non-hydrogenated and hydrogenated alloys. It can be seen that the *P*_max_ values for the non-hydrogenated as-built, HIP, and HT alloys are similar (1327 ± 56 N, 1365 ± 38 N, and 1293 ± 45 N, respectively). This observation apparently contradicts the results that reported by Ganor et al. [18], where the as-built yield stress was higher than that of the HIP alloy at 920 °C and HT at 1000 °C. The apparent discrepancy between the tensile test results in [18] and the current SPT results can be attributed to the differences in the loading direction. The tensile tests in [18] were conducted in the EBM alloy’s build direction, while the SPT experiments were conducted in the transverse direction (perpendicular to the EBM alloy’s build direction). The loading direction has a significant impact on the results, particularly in non-isotropic materials such as EBM alloys. Therefore, it is likely that the lack of correlation between the two methods is due to the differences in the loading direction [74]. The *P*_max_ values of all hydrogenated alloys decreased significantly, though the as-built hydrogenated alloy showed smaller degradation than either the HIP or HT alloy (a decrease of ~46%, ~65%, and ~64%, respectively).

Figure 10b displays the displacement at maximum load (*δ*_max_). It is evident that the maximal *δ*_max_ values of the non-hydrogenated alloys are similar, with an apparent increase after secondary processes (HIP alloy is higher). The elongation increase after secondary processes of the EBM alloy is in line with the trends reported by Ganor et al. [18]. After hydrogenation, *δ*_max_ was reduced significantly for all alloys, though the as-built hydrogenated alloy showed smaller reduction compared to both the HIP and HT alloys (a decrease of ~57%, ~77%, and ~76%, respectively).

Figure 10c displays the normalized energy (i.e., the area under the load-displacement curve at *P*_max_ divided by the specimen thickness) of non-hydrogenated and hydrogenated alloys. One can see that the normalized energy behavior supports the similarity in *P*_max_ and *δ*_max_ values of the non-hydrogenated alloys. The normalized energy of the hydrogenated alloys significantly decreased, though the as-built alloy had a smaller decrease compared to the hydrogenated HIP and HT alloys, in line with the trends in *P*_max_ and *δ*_max_.

The higher mechanical degradation of the hydrogenated HIP and HT alloys is attributed to their higher hydrogen content compared to the as-built alloy. This promoted microcrack formation within the bulk, likely resulting in early mechanical failure by HE mechanisms [72].

### 3.6. Fractography

**Figure 11:**
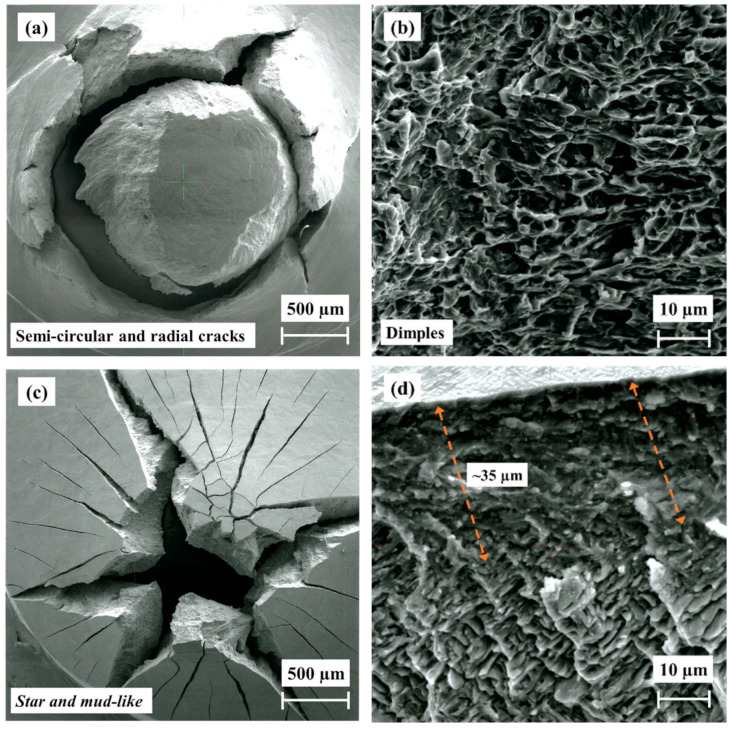
SEM fractography of as-built EBM Ti–6Al–4V alloy after SPT. (**a**,**b**) Non-hydrogenated; (**c**,**d**) hydrogenated.

The fracture (SEM observations) of non-hydrogenated and hydrogenated as-built EBM alloy following SPT is shown in Figure 11. The morphology of the non-hydrogenated as-built alloy, shown in Figure 11a, is semicircular with radial cracks extending out of the circle, indicating a mixed mode of ductile and brittle fracture. The fracture is characterized by dimples, as shown in Figure 11b, in agreement with previous observations by Lulu-Bitton et al. [23,24]. Figure 11b was acquired from an area close to the surface; it is similar to the morphology observed in the bulk area. Following hydrogenation, as shown in Figure 11c,d, the mode of fracture depicts a combination of radial (star-like) and mud-like cracks. The cross-sectional view close to the surface (Figure 11d) shows that the surface of the specimen contains a hydride layer with a thickness of ~35 µm. This layer exhibits a brittle behavior. Therefore, cracks are formed on the surface due to SPT loading and propagate through the brittle layer into the bulk; they stop at the interface between the brittle layer and the ductile bulk alloy beneath it. This fracture morphology is in agreement with our previous observation [23].

**Figure 12:**
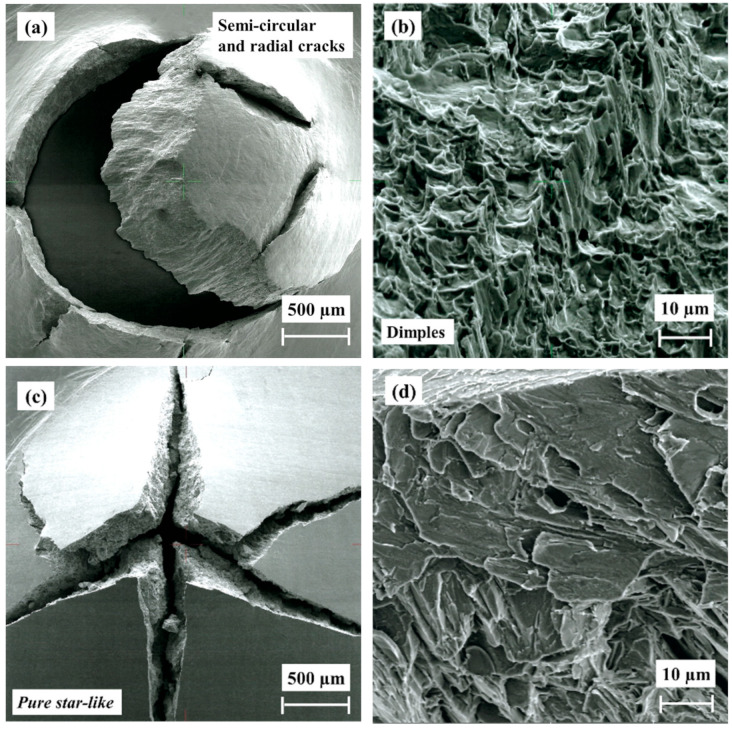
SEM fractography of HIP EBM Ti–6Al–4V alloy after SPT. (**a**,**b**) Non-hydrogenated; (**c**,**d**) hydrogenated.

The fracture surfaces of non-hydrogenated and hydrogenated HIP alloys are shown in Figure 12. The top-view fracture of the non-hydrogenated HIP alloy is semicircular with radial cracks (Figure 12a,b), similar to that observed for the non-hydrogenated as-built alloy. The fracture zone shown in Figure 12b, acquired from an area close to the surface, indicates the presence of dimples, i.e., ductile fracture. A similar morphology was observed in the bulk area. The top-view fracture of the hydrogenated HIP alloy is shown in Figure 12c,d. This fracture exhibits a pure star-like morphology, indicating a brittle fracture. This fracture is different from that observed in the hydrogenated as-built alloy, as it does not contain a mud-like texture. High magnification imaging (Figure 12d) acquired from an area close to the surface depicts cleavage fracture without any dimples.

**Figure 13:**
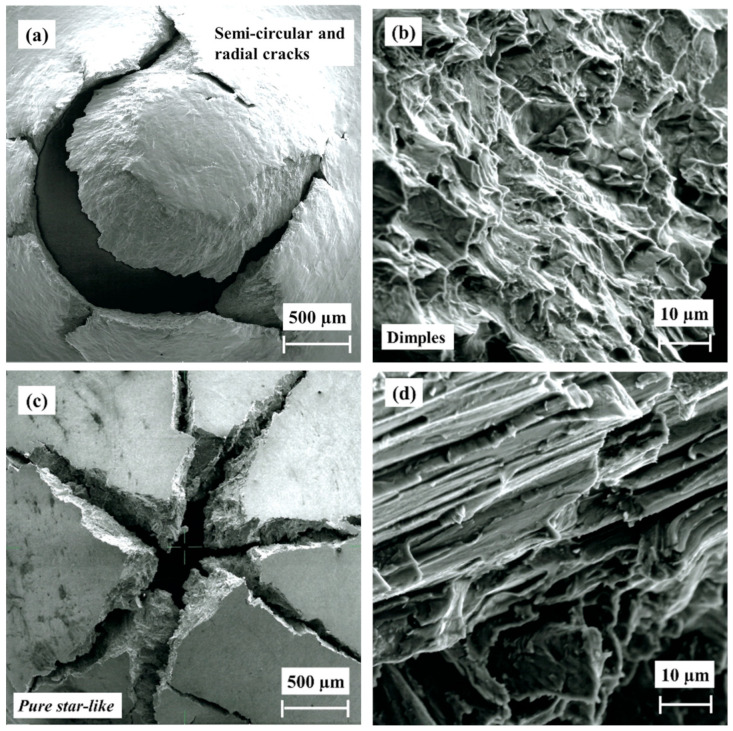
SEM fractography of HT EBM Ti–6Al–4V alloy after SPT. (**a**,**b**) Non-hydrogenated; (**c**,**d**) hydrogenated.

The fractography of the non-hydrogenated and hydrogenated HT alloy is shown in Figure 13. The top view of the non-hydrogenated HT alloy fracture is semicircular with radial cracks (Figure 13a), while the in-fracture view acquired from an area close to the surface shows the presence of dimples (Figure 13b), similar to those observed in the as-built and HIP non-hydrogenated alloys. A similar morphology was observed in the bulk area. The fracture of the hydrogenated HT alloy is a pure star-like morphology, indicating a brittle fracture (Figure 13c). This fracture is similar to that observed in the HIP hydrogenated alloy (Figure 12c). Figure 13d, acquired from an area close to the surface, shows a pure cleavage fracture without any dimples, which emphasizes the brittle nature of the fracture.

According to these results, the mode of fracture of the as-built alloy changed from ductile to mixed ductile and brittle following hydrogenation, while the fracture of the HIP or HT alloys changed after hydrogenation from ductile to pure brittle.

It can be concluded, that according to the findings, it is apparent that adhering to the process parameters specified in the ASTM F3301 standard for the HIP of EBM Ti–6Al–4V and the HT applied in the current study resulted in increased susceptibility to hydrogen adsorption after EC due to microstructural alterations. Thus, secondary processing parameter optimization is important in order to maintain the original EBM microstructure when HE is expected due to EC, for example, by reducing the specified ASTM HIP temperature of 920 °C for EBM Ti–6Al–4V.

## 4. Conclusions

The influence of electrochemical hydrogenation on the microstructure and mechanical behavior of EBM Ti–6Al–4V in the as-built, HIP, and HT thermo-mechanical conditions was studied. The non-hydrogenated alloys contained dual α and β phases and similar β-phase content, impurities, and densities. The hydrogenated alloys contained α/α_H_ solid solution, δ_a_ + δ_b_ hydrides, and a similar content of β_H_ solid solution. The main conclusions are listed below:

1. Secondary processes after EBM, such as HIP for 2 h at a temperature of 920 °C and a pressure of 120 MPa, or HT for 2 h in vacuum at a temperature of 1000 °C, change the β-phase morphology from discontinuous Widmanstätten to a more continuous β-phase morphology.
2. All three alloy conditions (as-built, HIP, and HT) exhibit a similar mechanical behavior in their non-hydrogenated state.
3. The fracture morphology of the hydrogenated HIP and HT alloys is similar, showing a pure brittle fracture with star-like morphology, while the as-built alloy shows a star-like and mud crack morphology due to the higher concentration of brittle hydrides in the outer surface.
4. Thermal/thermomechanical secondary processes of EBM Ti–6Al–4V alloy that promote an increase in the continuous β-phase morphology prior to EC are likely to make the alloy more susceptible to hydrogen embrittlement. The continuous β-phase morphology prevents hydride layer formation on the alloy’s surface, therefore enhancing hydrogen penetration into the bulk and increasing the alloy’s susceptibility to hydrogen embrittlement, compared to the as-built condition.
5. In applications involving EBM Ti–6Al–4V alloys in electrochemical hydrogen environments, including biomedical and aircraft applications, it is advisable to apply specific secondary treatment process parameters that prevent the increase of continuous β-phase morphology.

## Figures and Tables

**Table 1:** Designation of the EBM Ti–6Al–4V and the secondary processes.

Alloy	Designation	Secondary Process	
		HIP	HT
EBM	As-built	none	none
HIP	As-built + HIP	2 h at 920 °C, 120 MPa [18]; Heating and cooling rates: 4 °C/min	none
HT	As-built + HT	none	2 h at 1000 °C; 10^−3^ mbar [18]

**Table 2:** Impurity concentrations (wppm) in the non-hydrogenated EBM Ti–6Al–4V alloys.

Element	As-Built	HIP	HT
C	119 ± 6	123 ± 14	106 ± 15
O	1528 ± 34	1581 ± 9	1856 ± 89
H	48 ± 4	83 ± 8	71 ± 7
N	391 ± 41	404 ± 52	319 ± 74
Total	2086 ± 85	2191 ± 83	2352 ± 185

**Table 3:** Lattice parameters of the non-hydrogenated and hydrogenated alloys.

Specimen	α/α_H_	β/β_H_	δ_a_	δ_b_
**Non-hydrogenated as-built**	*a* = 2.924 Å *c* = 4.668 Å	*a* = 3.192 Å	-	-
**Non-hydrogenated HIP**	*a* = 2.925 Å *c* = 4.669 Å	*a* = 3.213 Å	-	-
**Non-hydrogenated HT**	*a* = 2.924 Å *c* = 4.669Å	*a* = 3.209 Å	-	-
**Hydrogenated as-built**	*a* = 2.922 Å, *c* = 4.682 Å	*a* = 3.260 Å	*a* = 4.323 Å	*a* = 4.148 Å
**Hydrogenated HIP**	*a* = 2.925 Å, *c* = 4.666 Å	*a* = 3.279 Å	*a* = 4.299 Å	*a* = 4.137 Å
**Hydrogenated HT**	*a* = 2.926 Å, *c* = 4.669 Å	*a* = 3.278 Å	*a* = 4.307 Å	*a* = 4.144 Å

**Table 4:** Maximum load, *P*_max_, displacement at maximum load, *δ*_max_ and normalized energy from SPT curves.

	Alloy	*P*_max_ [N]	*δ*_max_ [mm]	E [J/mm]
Non- hydrogenated	EBM	1327.6 ± 56.8	0.815 ± 0.065	1.56 ± 0.17
	HIP	1365.6 ± 35.8	0.893 ± 0.056	1.73 ± 0.23
	HT	1293.3 ± 45.7	0.933 ± 0.045	1.67 ± 0.19
Hydrogenated	EBM	711.3 ± 50.8	0.351 ± 0.035	0.37 ± 0.10
	HIP	474.6 ± 39.0	0.207 ± 0.022	0.13 ± 0.02
	HT	460.6 ± 43.4	0.221 ± 0.020	0.14 ± 0.03

## Data Availability

The data presented in this study are available on request from the corresponding author due to funding agency policy.
